# In vitro selection of *Staphylococcus aureus* mutants resistant to tigecycline with intermediate susceptibility to vancomycin

**DOI:** 10.1186/s12941-016-0131-7

**Published:** 2016-03-08

**Authors:** Melina Herrera, Sabrina Di Gregorio, Silvina Fernandez, Graciela Posse, Marta Mollerach, José Di Conza

**Affiliations:** Facultad de Ciencias de la Salud, Universidad Adventista del Plata, 25 de mayo 99, Libertador San Martín, Entre Ríos Argentina; Facultad de Farmacia y Bioquímica, Universidad de Buenos Aires, Junín 956, Ciudad Autónoma de Buenos Aires, Argentina; Facultad de Bioquímica y Ciencias Biológicas, Universidad Nacional del Litoral, Ciudad Universitaria, Paraje “El Pozo”, CC 242, Santa Fe, Argentina

**Keywords:** MRSA, Tigecycline resistant, Efflux activity, VISA

## Abstract

**Background:**

Tigecycline (TIG) is an antibiotic belonging to the glycylcyclines class and appears to be a good choice to fight infections caused by *Staphylococcus aureus*. To date, TIG exhibits good activity against this microorganism. The aim of this work was to obtain in vitro mutants of *S. aureus* resistant to TIG and evaluate possible changes in their susceptibility patterns to other antibiotics.

**Results:**

Two mutants of *S. aureus* resistant to TIG (MIC = 16 µg/mL) were selected in vitro from clinical isolates of methicillin-resistant *S. aureus*. In both mutants, corresponding to different lineage (ST5 and ST239), an increase of efflux activity against TIG was detected. One mutant also showed a reduced susceptibility to vancomycin, corresponding to the VISA phenotype (MIC = 4 µg/mL), with a loss of functionality of the *agr* locus. The emergence of the VISA phenotype was accompanied by an increase in oxacillin and cefoxitin MICs.

**Conclusions:**

This study demonstrates that, under selective pressure, the increase of efflux activity in *S. aureus* is one of the mechanisms that may be involved in the emergence of tigecycline resistance. The emergence of this phenotype may eventually be associated to changes in susceptibility to other antibiotics such oxacillin and vancomycin.

## Findings

*Staphylococcus aureus* is one of the major pathogens causing serious infections both within the hospital setting and in the community. This pathogen is characterized by rapid acquisition of resistance to antibiotics introduced into clinical practice. Thus, methicillin-resistant *S. aureus* (MRSA) emerged first in the hospital setting and then spread to the community (CA-MRSA) [1]. In the late 1990s, MRSA strains emerged with reduced susceptibility to vancomycin, VISA (vancomycin-intermediate *S. aureus*) [2] and VRSA (vancomycin-resistant *S. aureus*) [3]. Tigecycline (TIG) is an antibiotic belonging to the glycylcyclines class and representing a treatment option for infections caused by *S. aureus* [4]. Surveillance studies of *S. aureus* have exhibited good activity of this antibiotic, with 99.9 % of isolates found to be susceptible [5]. A high susceptibility rate was also reported in Latin America from 2004 to 2010 [6] and in several countries around the world [7, 8]. The aim of this work was to select and characterize in vitro tigecycline-resistant mutants from MRSA clinical isolates.

Two unrelated MRSA clinical isolates (2028p and 94159p) were studied. They were genotyped by *spa* typing [9], and the multilocus sequence type (MLST) was determined using the *S. aureus* MLST database (http://www.mlst.net).

Oxacillin resistance was confirmed by PCR amplification of an internal fragment of the *mecA* gene. *S. aureus* strains ATCC 29213 and ATCC 43300 were used as negative and positive controls, respectively.

The SCC*mec* type was determined by characterization of the *ccr* complex (cassette chromosome recombinase) and the *mec* complex, using a simplified version of the previously described scheme [10]. The *agr* type was characterized by multiplex PCR [11], and analysis of *agr* functionality was performed by determining δ-hemolysin production according to Traber et al. [12]. Briefly, it was conducted by cross-streaking test strains perpendicularly to *S. aureus* RN4220, which only produces β-hemolysin on a sheep blood agar plate. δ-hemolysin acts synergistically in the lysis of sheep red blood cells and generates a zone of enhanced hemolysis at the intersection of RN4220 and test strain streaks.

In vitro mutant selection was performed by serial passage in Mueller–Hinton broth (Britania, Argentina) with increasing concentrations of TIG (Pfizer, USA), starting from a sub-inhibitory concentration corresponding to ¼ minimum inhibitory concentration (MIC) to MIC values, using an inoculum of 5 × 10^5^ CFU/mL. Colonies were selected after 15 passages [13]. The MIC of TIG was determined by the epsilometric method, considering the FDA breakpoints. Mutant stability was evaluated by determining the TIG MIC after 10 consecutive passages in antibiotic-free Tryptic-Soy Agar (Britania, Argentina). The clonal relationship between the parental strains and the mutants was confirmed by pulsed-field gel electrophoresis (PFGE) using the *Sma*I endonuclease [14]. Susceptibility to other classes of antibiotics was tested by the agar dilution method following the Clinical and Laboratory Standards Institute recommendations (CLSI, 2013). The antibiotics tested were oxacillin (OXA), cefoxitin (FOX), trimethoprim-sulfamethoxazole (TMS), rifampicin (RIF) (Sigma-Aldrich, USA), gentamicin (GEN), ciprofloxacin (CIP), clindamycin (CLI) and vancomycin (VAN) (Fada Pharma, Argentina).

Finally, efflux activity was phenotypically evaluated as a potential mechanism of resistance to TIG by comparing the MICs of TIG and ethidium bromide (EB) in the presence and absence of reserpine (RS) (20 µg/mL). An EB MIC of ≥32 µg/mL, coupled with a reduction of at least 4 twofold dilutions (TFD) in the MICs of EB and TIG in the presence of RS, was considered to be indicative of an enhancement of efflux activity. This criterion combines the canons proposed by Patel et al. [15] (EB MIC ≥25 µg/mL) and DeMarco et al. [16] (MIC reduction of 4 TFD in the presence of RS).

**Fig. 1:**
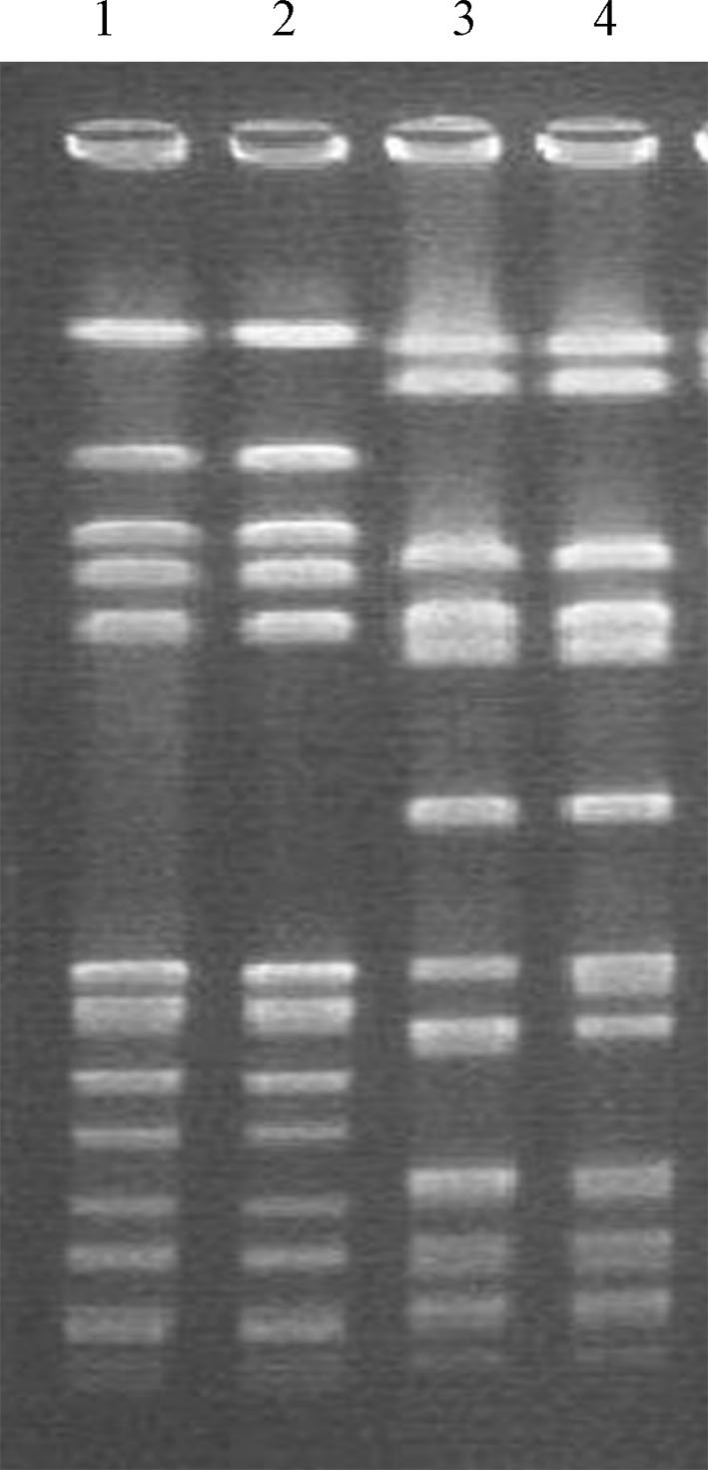
*Sma*I-PFGE of the parental and mutant strains. *Lane 1*: parental strain 2028p, *lane 2*: mutant strain 2028 m, *lane 3*: parental strain 94159p, *lane 4*: mutant strain 94159 m

The two TIG-resistant mutants were obtained from the two MRSA parental strains, 2028 and 94159p, and named 2028 and 94159m, respectively. The parental and mutant strains were isogenic (Fig. 1). Both mutants exhibited TIG MIC values, which were 128-fold higher than those against the parental strains. The MIC data and molecular characteristics of the strains are summarized in Table 1.

**Table 1:** Molecular and phenotypic characterization of two TIG-resistant mutants and MRSA parental strains

Strains	Molecular characterization				MIC (μg/mL)															δ-hemolysin
	SCC *mec*	ST	*spa* type	*agr* group	OXA	FOX	VAN	TMS	RIF	CIP	GEN	CLI	TIG	TIG + RS	EB	EB + RS	OXA + RS	FOX + RS	VAN + RES	
2028p	III	239	t654	I	≥32	≥128	1	≥32/608	≥16	≥16	≥64	≥8	0125	0.064	16	2	ND	ND	ND	+
2028m	III	239	t654	I	≥32	≥128	1	≥32/608	≥16	≥16	≥64	≥8	16	0.25	64	2	ND	ND	ND	+
94159p	IV	5	t002	II	8	16	1	0.5/9.5	4	0.5	≥64	≤0.25	0125	0.064	16	1	16	16	1	+
94159m	IV	5	t002	II	32	64	4	0.5/9.5	4	0.5	≥64	≤0.25	16	1	128	8	32	64	4	–

*p* parental, *m* mutant, *ST* sequence type, *OXA* oxacillin, *FOX* cefoxitin, *VAN* vancomycin, *TMS* trimethoprim sulfamethoxazole, *RIF* rifampicin, *GEN* gentamicin, *CLI* clindamycin, *TIG* tigecycline, *RS* reserpine, *EB* ethidium bromide, *ND* not determined

Unlike the parental strains, both mutants showed a decrease of ≥4 TFD in EB and TIG MICs in the presence of RS (Table 1), which suggested that an increase in efflux pump activity could be involved in TIG resistance. It is well known that efflux pumps in *S. aureus* have the ability to expel more than a few antibiotics in addition to other compounds such as biocides and dyes [17]. The increase of efflux activity is one of the mechanisms involved in resistance of *S. aureus* to several antibiotics, due to which strains become refractory to treatments with those antibiotics [18].

To date, naturally occurring *S. aureus* isolates with reduced susceptibility to tigecycline (MICs of 1–2 µg/mL) have been isolated from clinical specimens [5, 6]. However, the high MIC values (16 µg/mL) of these in vitro selected mutants should be considered a potential risk in clinical settings. It is important to highlight that no significant fitness cost associated with the selection of these mutants was detected (data not shown).

In addition, TIG-resistant mutant 94159 m was also characterized by a changed susceptibility profile to OXA, FOX, and VAN. It is important to highlight that the VAN MIC value of this mutant is 4 µg/mL, thus corresponding to the VISA definition (Table 1).

An increase in the VAN MIC was previously associated with a reduction in the OXA MIC in both in vitro selected VRSA mutants and in vivo VISA isolates [19, 20]. By contrast, in this case the emergence of the VISA phenotype is accompanied by an increase in OXA and FOX MIC values in the 94159m strain.

The MIC values of OXA, FOX and VAN for 94159m remain unchanged in the presence of RS (Table 1) suggesting a different mechanism to that observed for TIG resistance.

**Fig. 2:**
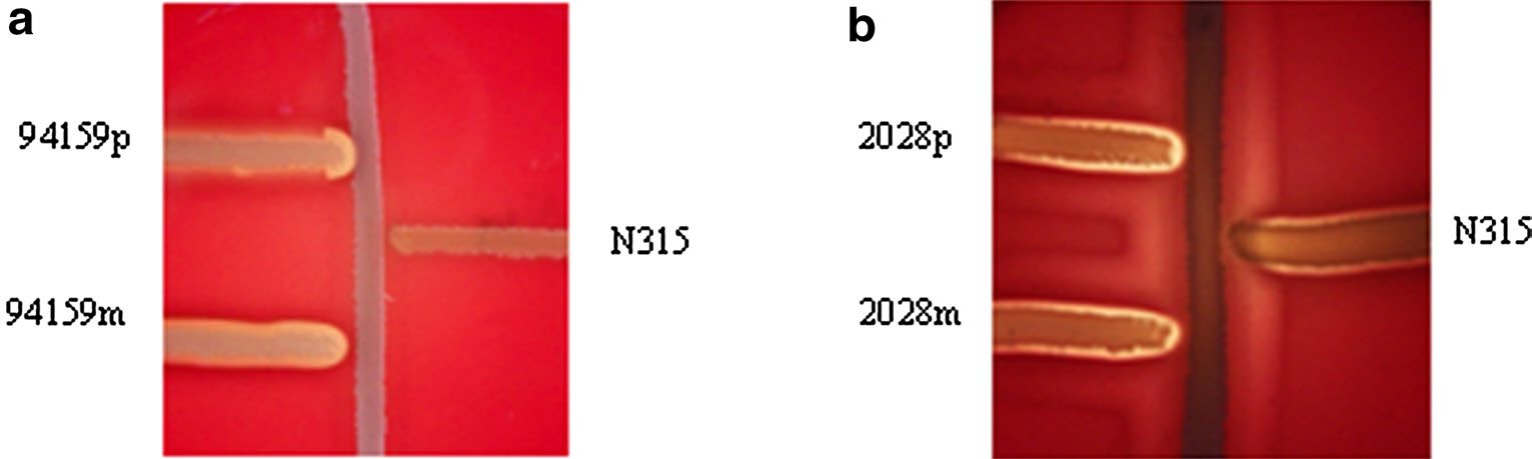
δ-Hemolysin assays. The strain streaked vertically is β-hemolysin-producing RN4220. Strain N315 was used as a negative control. **a** A zone of enhanced hemolysis by strain 94159p is seen as an *arrowhead* (δ-hemolysin); the δ-hemolytic capacity has been lost by strain 94159 m. **b** Strains 2028p and 2028 m both produce β- and δ-hemolysins

Based on molecular typing, the 94159p strain was characterized as ST5, SCC*mec* IV, *spa*-type t002, indicating that it belonged to the main CA-MRSA clone that circulated in Argentina at the time when this strain was isolated [21, 22]. The increased ability to acquire new resistance determinants and the capacity of surviving in different environments have been associated with a great genomic plasticity of clonal complex 5 (CC5). The majority of heterogeneous VISA (hVISA), VISA and VRSA isolates belong to this lineage [23, 24]. Likewise, an emergence of CC5 hVISA isolates has recently been reported in Argentina [25]. Finally, a loss of δ-hemolysin expression in the VISA 94159m mutant was another characteristic observed in this work (Fig. 2). An association between reduced susceptibility to VAN and the loss of the *agr* function was described previously [26].

Strain 2028p (ST239, SCC*mec* III, *spa*-type t654) was shown to belong to the Brazilian clone, a multi-resistant HA-MRSA clone that was prevalent in Argentina in 2005. Contrary to the behavior of 94159m, mutant 2028m did not show any modification in either the VAN MIC or in the *agr* functionality. In this work, the ability of *S. aureus* to develop resistance to TIG under selective pressure with this antibiotic was shown, and the increase of efflux activity is considered to be one of the possible resistance mechanisms involved. The selection of TIG mutants in two different lineages indicates that this event is not limited to a particular genetic background. Furthermore, the data show that, in a particular strain, the acquisition of this resistance may be associated with reduced susceptibilities to vancomycin and some other antibiotics such as oxacillin. The literature data suggest that the phenomenon of elevated vancomycin MICs, coupled with the loss of δ-hemolysin expression, appears to be common to different geographical regions [27]. Importantly, while the emergence of resistance to tigecycline and vancomycin can occur, the absence of high-level resistance to these antibiotics is noteworthy [5]. It is important to be aware of this potential risk and, wherever possible, emphasize the necessity to use appropriate and adequate drug dosing regimens to prevent it.

## Availability of supporting data

The data set supporting the results of this article is included within the article.
